# A probabilistic health risk assessment of potentially toxic elements in edible vegetable oils consumed in Hamadan, Iran

**DOI:** 10.1186/s12889-023-17624-1

**Published:** 2024-01-18

**Authors:** Fereshteh Mehri, Ali Heshmati, Elaheh Talebi Ghane, Mohammad Khazaei, Trias Mahmudiono, Yadolah Fakhri

**Affiliations:** 1 Nutrition Health Research Center, Hamadan University of Medical Sciences, Hamadan, Iran; 2 Modeling of Noncommunicable Diseases Research Center, Hamadan University of Medical Sciences, Hamadan, Iran; 3 Department of Environmental Health Engineering, Research Center for Health Sciences, Hamadan University of Medical Sciences, Hamadan, Iran; 4 Department of Nutrition, Faculty of Public Health, Universitas Airlangga, Jl. Mulyorejo Kampus C, Surabaya, 60115 Indonesia; 5 Food Health Research Center, Hormozgan University of Medical Sciences, Bandar Abbas, Iran

**Keywords:** Food security, Potentially toxic element(s), Edible vegetable oils, Health risk, Monte Carlo simulation

## Abstract

In this study, potential toxic element (PTEs) including lead (Pb), arsenic (As), cadmium(Cd), iron (Fe) and zinc (Zn) in traditional and industrial edible vegetable oils (peanut, sunflower, olive and sesame) collected from Hamadan, west of Iran were determined using Inductivity Coupled Plasma Optical Emission Spectrometry (ICP-OES). Besides, probabilistic health risk assessment (non-carcinogenic and carcinogenic risks) was identified via total target hazard quotient (TTHQ) and cancer risk (CR) by the Monte Carlo Simulation (MCS) model. The ranking of concentration PTEs in traditional and industrial edible vegetable oils was Fe > Zn > As > Pb > Cd. The in all samples, content of PTEs in industrial oils were upper than traditional oils (*p* < 0.001). The level of PTEs in most of vegetable oils was lower than permissible concentration regulated by Codex and national standard. In term of non-carcinogenic, consumers were at acceptable range (TTHQ < 1) due to ingestion both traditional and industrial vegetable oils content of PTEs. In term of carcinogenic, CR the both adults and children was higher than acceptable range (CR < 1E-6), Hence consumer are at unacceptable risk due to ingestion industrial vegetable oils content of inorganic As. Therefore, it is recommended to implement control plans for PTEs in vegetable oils consumed in Hamadan, Iran.

**Supplementary Information:**

The online version contains supplementary material available at 10.1186/s12889-023-17624-1.

## Introduction

Food security is one of the chief global worries in the last years [1–7]. Environmental pollution in soil [8, 9], air [10–13], water resources [14, 15], food such as dough, meat, wheat, rice and etc [1, 2, 16–30] has increased over decades [23, 24, 28, 31]. These pollutants includes microbial [32–34], mycotoxins [5, 35–38], heavy metals [6, 32, 39–47], pesticides, antibiotics [8, 48] and other contaminants [49–53] caused cancers [54–58] and other diseases [59–61]. The demand for the using of vegetable oils in different fields such as chemical science, medicinal, and cosmetic and active industries has increased in various counties of worldwide in recent decade [62].

The as reported during 2001–2011, mean per capita consumption of oils was stated to be 10.71 kg [63]. Vegetable oils are water insoluble constituents obtained from plants, which contain chiefly of long-chain fatty acid esters derived from the alcohol glycerol [64]. These compounds due to contain different components including linoleic, stearic, oleic, protein, carbohydrate, palmitic acid, antioxidant and vitamins play important role in human health as production energy source, use in structural component and help to absorption of the fat-soluble vitamins in body human [65, 66]. Moreover consumption of oils can result to cholesterol reduction effect, and prevention of cardiovascular pathologies [67].

However, many reports have documented the occurrence of potentially toxic elements in edible vegetable oils and soil in different regions of Iran and world [68–70] although there is a little information in Hamadan province, the western region of Iran.

The important objectives of the current study are 1) to determine the concentration of PTEs in the vegetable oils (sunflower, peanut, sesame and olive)available in the western Iran market using Inductivity Coupled Plasma Optical Emission Spectrometry (ICP-OES) detection, 2) to compare the occurrence levels compared to European Union and Iranian Standards maximum limits, 3) to assess the risks to the consumers exposure to PTEs in vegetable oils in Iran, 4) to draw the necessary consideration of the public health authorities towards conducting extensive monitoring and establish regulations for PTEs. The results of the current study will be beneficial for farmers, merchants, and consumers in the community.

## Material and method

### Collection of samples

The twenty of traditional vegetable oil including peanut (*n* = 5), sunflower (*n* = 5), olive (*n* = 5) and sesame (*n* = 5) were completely random bought from local shop and farmer’s market in Hamadan, western Iran. Moreover, the twenty of vegetable oil samples peanut (*n* = 5), sunflower (*n* = 5), olive (*n* = 5) and sesame (*n* = 5) were obtained from the various cities of the province as industrial sample during 2022. All samples kept in closed polyethylene containers were stored in the temperature (4 °C) until the examination time.

### Chemical materials, digestion samples and method validation

All solutions were purchased from Merck, (Darmstadt, Germany). Double de-ionized water was applied for all dilutions. Stock standard solutions of all elements (1000 mg/L) were prepared from Merck was used for calibration standards. HNO_3_ (65%), de-ionized water, and H_2_O_2_ (30%) with a grade of spectroscopic, was taken by Merck (Darmstadt, Germany). All the Falcons pipe were washed by drenching in a nitric acid solution (10%) and formerly washed with de-ionized water prior to experimental to reduce the pollution of sample. For digestion 1 mL of each sample was poured in a glassware and were assimilated with 6 ml of HNO_3_ (65%) and 2 ml of H_2_O_2_ (30%) and then the sample was stirred at room temperature for 11 h. The time and power of digestion was set in two stages—stage 1 (1 h at 80 °C) and stage 2 (3 h at 150 °C) [66]. In the continuation of the experiment, all samples were transferred to vials of ICP-OES for examination. Simultaneously, blank samples were organized with the same technique.

## Method validation

The validation for PTEs in samples was done based on the procedure study [71]. The content of LOD and LOQ were considered using 3 and 5 times the standard deviation of the reply on the standard curve slope (SD/b), respectively. The linearity, recovery percentages, regression coefficient (R^2^), LOQ and LOD, of metals in various samples are presented in Table 1.

**Table 1:** Method validation parameters reported by ICP-OES analysis in vegetable oils

Metals	Wavelength	R^2^ Value	Calibration Range	Recovery %	LOD (µg/L)	LOQ (µg/L)
As	188.03	0.998	0.2–1400	99	0.16	0.50
Cd	227.80	0.997	0.3–1400	96	0.02	0.09
Pb	282.30	0.994	1.4–1400	99	0.12	0.41
Fe	328.2	0.996	0.4–1300	100	0.63	1.89
Zn	204.19	0.999	0.3–1300	95	0.43	1.30

### Determination of PTEs

The measure of all vegetable oil samples was done by ICP-OES techniques. The operating factors were including: producer of RF was1400 W; the argon gas applied for plasma and nebulizer auxiliary was. Gas flow used for plasma, auxiliary, and nebulizer were 14.50, 0.90 and 0.85 Lmin^−1^, respectively. Gas flow of 14.50, 0.90 and 0.85 Lmin^−1^, were utilized for plasma, auxiliary, and nebulizer respectively. Moreover, period of uptake, rinse, and initial stabilization of samples was 240, 45 and 45 s, respectively. Also, period of replicate were zero. The frequency of the producer of RF was 27.12 MHz. Cyclonic and modified Lichte, was used as the types of detector solid state and spray chamber respectively. The delivery pump of sample was used four-channel, Furthermore the speed of pre-wash pump (rpm) was set as 60 (for 15 s), 30 (for 30 s), at the ultimately, the injection pump speed and time of pre-wash was 30 rpm and 45 s respectively.

### Probabilistic health risk assessment

#### Non-carcinogenic risk

The chronic daily intake (CDI), target hazard quotient (THQ) and total target hazard quotient (TTHQ) due to ingestion vegetable oils content of PTEs were calculated based on the following equations [72–75].

1 $$\mathrm{CDI }= \frac{\mathrm{C }\times \mathrm{IR }\times \mathrm{ED }\times {\text{EF}}}{\mathrm{BW }\times {\text{AT}}}$$

2 $$\mathrm{THQ}=\frac{\text{CDI}}{\mathrm{RfD}\;\mathrm{or}\;\mathrm{TDI}}$$

Where, CDI is chronic daily intake; C, concentration of PTEs (mg/kg); IR, ingestion rate; EF, exposure frequency (350 days/year); ED, exposure duration that for children and adults was set 6 and 70 years, respectively; BW, body weight that for children and adults was set 15 and 70 kg, respectively; and AT for non-carcinogenic risk is the mean time life span for children and adults is equal to 2190 and 25550 days, respectively and for carcinogenic risk for both children and adults is equal to 25550 days. IR for vegetable oil by Iranian consumers which was set at (adults (100 g/d-n) and children (20 g/d-n)), respectively [76]. Oral RfD indication oral reference dose of set for Cd, Zn, Fe and As was 0.001, 0.3, 0.7 and 0.0003 mg/kg-d, respectively. While, TDI for Pb was 0.0036 mg/kg-d [62].

3 $$\mathrm{TTHQ }= {\sum }_{{\text{J}}}^{{\text{j}}}{\text{THQ}}$$

Where, TTHQ is sum of individual THQ. When THQ and/or TTHQ is lower than 1 value consumers are at safe non-carcinogenic risk [62].

#### Carcinogenic risk

The carcinogenic risk (CR) of the inorganic As in vegetable oils was calculated using this equation

4 $$\mathrm{CR }=\mathrm{CDI }\times {\text{CSF}}$$

Where, CSF is cancer slope factor that is for inorganic As 1.5 (mg/kg-d)^−1^. When CR is lower than 1.00E-6 value, consumers are at safe carcinogenic risk [62].

### Uncertainty analysis

Then single-point values were employed for evaluation of risk, high uncertainty can occur in the analysis of the results, therefore for raise precise of risk Monte Carlo simulated (MCS) technique was utilized for estimate health risk. The type of distribution of PTEs concentration data was obtained as log-normal through fit-distribution command and according to similar studies distribution of ingestion rate was selected log-normal [77–79]. In this way cut point of risk was considered as median of THQ and CR with 50,000 repetitions [80].

### Statistical analysis of study

The measurement of all data was conducted by the SPSS 11.5.1 software. A finding was indicated in three repetitions and was indicated as mean ± standard deviation (M ± SD). The difference among the PTEs was stated by one-way ANOVA, and the Duncan’s new multiple was used as post-doc test. The significance was set at 0.05.

## Results and discussion

### PTEs concentration in traditional edible vegetables oils and comparison with published studies

In this study, mean content of PTEs (As, Cd, Pb, Fe, and Zn) in 40 samples of traditional and industrial edible vegetables oils were shown in Table 2. As seen of results, there were suitable linear associations between the answers of instrumental and the PTEs level in edible oils with correlation coefficients of 0.997 or upper. The optimized method indicated to have the high sensitive and selective, so that LOD, LOQ and recover was ranged from (0.02 to 0.63 µg/L), (0.09 to 1.89µg/L) and (95% to 100%), respectively Table 1. According to results, as seen from Table 2, A mean concentration of 0.60 ± 0.22 (As), 0.005 ± 0.02 (Cd), 8.92 ± 0.91 (Fe), 0.14 ± 0.004 (Pb), and 3.74 ± 0.53 (Zn) mg/kg was reported for olive oil samples. With regards to sunflower oil samples, mean concentration of As, Cd, Fe, Pb, and Zn was 0.05, 0.011, 2.19, 0.011, and 2.10 mg/kg, respectively (Table 2). Moreover, mean concentration of As, Cd, Fe, Pb, and Zn in sesame oil samples was 0.06, 0.009, 15.09, 0.009 and 3.19mg/kg, respectively. These values in peanut oil samples were 0.005, 0.006, 5.65.0.25 and 2.86 mg/kg respectively.

**Table 2:** Mean concentration of PTEs (mg/kg) in traditional and industrial vegetable oils samples (*n* = 40)

**Oil**		**As**	**Cd**	**Fe**	**Pb**	**Zn**
**Olive**	**Traditional**	0.060 ± 0.022	0.005 ± 0.002	8.928 ± 0.913	0.014 ± 0.004	3.756 ± 0.538
	**Industrial**	0.113 ± 0.028	.007 ± 0.002	11.739 ± 2.147	0.016 ± 0.002	3.644 ± 0.458
	***P*-value**	< 0.001	0.045	< 0.001	0.119	0.544
**Sunflower**	**Traditional**	0.052 ± 0.032	0.011 ± 0.006	2.195 ± 1.226	0.011 ± 0.003	2.102 ± 0.654
	**Industrial**	0.053 ± 0.021	0.010 ± 0.004	0.097 ± 0.028	0.016 ± 0.003	3.551 ± 0.542
	***P*-value**	0.946	0.827	< 0.001	< 0.001	< 0.001
**Sesame**	**Traditional**	0.064 ± 0.037	0.009 ± 0.004	15.091 ± 1.182	0.009 ± 0.004	3.192 ± 2.143
	**Industrial**	0.091 ± 0.033	0.009 ± 0.003	23.664 ± 2.698	0.013 ± 0.002	6.299 ± 0.974
	***P*-value**	0.043	0.603	< 0.001	< 0.001	< 0.001
**Peanut**	**Traditional**	0.005 ± .003	0.006 ± .002	5.655 ± 1.477	0.025 ± 0.035	2.863 ± 1.977
	**Industrial**	0.089 ± .038	0.009 ± .002	11.323 ± 1.726	0.027 ± .045	8.835 ± 1.346
	***P*-value**	< 0.001	< 0.001	< 0.001	0.889	< 0.001
**Total**	**Traditional**	0.046 ± .035	0.008 ± 0.004	7.967 ± 4.937	0.015 ± 0.018	2.978 ± 1.598
	**Industrial**	0.086 ± .037	0.009 ± 0.003	11.706 ± 8.613	0.018 ± 0.023	5.582 ± 2.366
	*P*-value	< 0.001	0.172	0.004	0.370	< 0.001
*P*-value	Type	< 0.001	0.124	< 0.001	0.356	< 0.001
	Oil	< 0.001	< 0.001	< 0.001	0.019	< 0.001
	Type × Source	0.201	0.078	0.389	0.985	0.113

Compared to our results, Heidary et al. indicated mean content of Cd, Pb, and Zn, in olive oil of Iran country was 0.071, 0.22 and 41.38 mg/kg, respectively [81]. In conducted study by Luka et al. on olive oil samples from the Cyprus country mean level of Cd, Pb and As was 0.08, 0.45 and 0.19 mg/kg than were upper than our study [82]. Ieggli et al. concluded mean concentration of Fe, Pb, As and Cd in sunflower oil samples of Saudi Arabia country was 99.3, 0.09, 0.08 and 0.09 mg/kg [83]. Consistent with this study, in research conducted by Zhu et al., mean concentration of Fe and Pb obtained 29.2 and 0.010 mg/kg [84] that was above our results. Acar et al. reported level contamination of Pb, Fe and Zn in Sunflower oil samples of Turkey country was 0.08, 1.51and 1.23 mg/kg that than our results was lower [85]. Yao et al. expressed mean concentration of Pb and Cd in peanut oil samples of China country was 0.093 and 3.47 µg kg-1 [86]. Asemave et al. indicated mean concentration of Fe, Cd and Pb in peanut oil samples of Nigeria country was 8.51, 0.16 and 0.02 µg kg-1[87]. Zhu et al. reported mean concentration of As, Pb, Cd, Fe, and Zn in sesame oil samples of China country was 0.015, 0.014, 0.005, 37.9 and 0.07 µg kg-1, respectively [84]. Razzaghi et al. indicated mean concentration of As, Pb, Cd, Fe, and Zn in sesame oil samples of Iran country was 0.011, 0.016, 0.001, 3.49 and 1.70 µg kg-1, respectively [88].

### PTEs concentration in industrial edible vegetables oil and comparison with published studies

In our study, 40 samples from various edible vegetable oils were investigated; of which 20 samples were industrial products. Mean concentration of As, Cd, Fe, Pb, and Zn, in industrial olive oil samples were 0.11 ± 0.02, 0.007 ± 0.002, 11.73 ± 2.14, 0.16 ± 0.002, and 3.64 ± 0.45 mg/kg, respectively (Table 2). With regards to results, mean concentration of 0.053 ± 0.02 (As), 0.010 ± 0.004 (Cd), 2.97 ± 0.28 (Fe), 0.16 ± 0.003 (Pb), and 3.51 ± 0.54 (Zn) mg/kg was detected for sunflower oil samples, respectively. Mean concentration of As, Cd, Fe, Pb, and Zn in sesame oil samples was 0.091, 0.009, 23.66, 0.013 and 6.29mg/kg, respectively. These values in industrial peanut oil samples were 0.86, 0.009, 11.32, 0.0.027 and 8.83 mg/kg respectively.

Aligned with our findings, Mendil et al. indicated concentration of PTEs such as Cd, Pb, Zn and Fe in industrial olive oils samples of Turkey country was 0.15, 0.03, 1.03 and 139.0 respectively that in comparison with our study amount of Cd and Zn was lower and Pb and Fe was higher [89]. Farzin et al. showed level of Cd and Pb in industrial olive oils samples of Iran country was 0.045 and 0.046 mg/kg respectively that was lower than this study [66]. In a similar study, Zhu et al. showed level of PTEs such as Cd, Pb, Zn and Fe in industrial olive oils samples of China country was 0.002, 0.009, 32.8 and 1.24 respectively [66]. Compared to our findings, Dugo et al., reported concentration range of Pb and Zn in industrial sunflower oil of Italy country was 0.005–0.016 and 0.08–0.33 mg/kg, respectively. Ashraf et al. showed mean concentration of As, Cd, Fe and Zn in industrial sunflower oil samples of Saudi Arabia country was 0.013, 0.002, 33.4, 0.011 and 1.54 mg/kg [90]. The results of these two studies were lower than our findings. Furthermore, Pehlivan et al. concluded mean concentration of Fe, Pb, As and Cd in industrial sunflower oil samples of Turkey country was 0,01, 0.002, 0.001 and 0.02 mg/kg [91]. Consistent with this study, Nunes et al., reported mean concentration of Fe and Pb in industrial sunflower oil samples of Brazil country obtained 2.99 and 0.3 mg/kg [92], that was higher compared to the results of this study. Zhu et al. reported mean concentration of As, Cd, Fe, Pb, and Zn in industrial sesame oil samples of China country was 0.019, 0.018, 0.005, 37.9 and 0.78 mg/kg, respectively. These values in industrial peanut oil samples were 0.013, 0.007, 0.003, 44.7 and 1.18 mg/kg, respectively [84]. Asemave et al. indicated mean concentration of As, Fe, Pb, and Cd in industrial peanut oil samples of Nigeria country was 8.51, 0.16, and 0.002 mg/kg, respectively [87]. In other study conducted by Ashraf et al., they reported concentration of As, Cd, Fe, Pb and Zn for industrial peanut oil samples was 0.017, 0.003, 37.8, 0.13 and 1.71 mg/kg, respectively. While, these values in industrial sesame oil was 0.018, 0.005, 43.8, 0.17 and 0.95 mg/kg, respectively [90].

### Comparison of PTEs in traditional and industrial edible vegetables oils with standard level

According to (Table 2), the PTEs such as Fe, Cd, As, Zn and Pb were identified in all traditional and industrial edible vegetables oils. PTEs content was obtained in order of Fe > Zn > As > Pb > Cd in traditional and industrial vegetables oils, respectively. The level of all PTEs in industrial oils were higher compared traditional oils (*p* <  < 0.001). The findings of statistical analysis indicated the oil varieties have significant effect the on concentration of PTEs except Cd and Pb. Though, sources (brands) and interaction of Type × Source did not express any effect on the amount PTEs. In our study, the content of PTEs from various edible oils was compared with international organizations code. In this study, concentration of PTEs in all samples was lesser relative to maximum concentration permissible of As (0.1 mg/kg), Cd (0.05 mg/kg), Pb (0.1 mg/kg) regulated by Codex and national standards [66]. All traditional and industrial oils samples concentration of Fe was higher than maximum permissible concentration of Fe (1.5 mg/kg) except industrial sunflower oil [66]. As seen from Table 1, level Zn in all samples was higher than Provisional Tolerable Daily Intakes PTDI (mg/kg-bw) Considered by Institute of Standards and Industrial Research of Iran (ISIRI) standards [81].

As mentioned in current study and in those of previous investigations, there is significant change between metal content in traditional and industrial oils samples. Based on studies, species and breed of the cultivated plant, difference in the bioavailability each of PTEs, improper use of fertilizers, near to roads with heavy traffic, industrial factories, active mines, highways, as well as, storage situation and processing equipment can be mentioned as influential factors [62]. In line with these differences, Nayek et al. reported high mobility of PTEs such as Fe and Cd than Pb can lead to transfer them from polluted soil to plant stems [93]. Ansari et al. concluded that the using of appropriate seeds and irrigation system, could decrease the metal value in sunflower oil [94]. In traditional oils samples, apply of various fertilizers in farming environment can alters the pH, organic level, and biodegradation of PTEs in the soil [95]. Similarly to this study, Ab Manan et al. indicated rise the absorption of PTEs can occur due to decrease of pH and formation of metal–carbonate complexes in oil producing plants [96]. In industrial oils samples, technologies utilized in the manufacture of oil by different processing (bleaching, hardening, refining, and deodorization) can effectively rise the amount of PTEs in raw compounds of seed plants [97]. Finster et al., reported concentration of Pb in the soils of parts near to tetraethyl emissions originated from exhausted car, was upper compared to the soil of other parts [76]. The weather conditions, pollution of the region the like (air pollution from industries, sewage from factories near cultivation) can be mentioned as other sources of contamination from PTEs in oils [65].

### Probabilistic health risk assessment

**Fig. 1:**
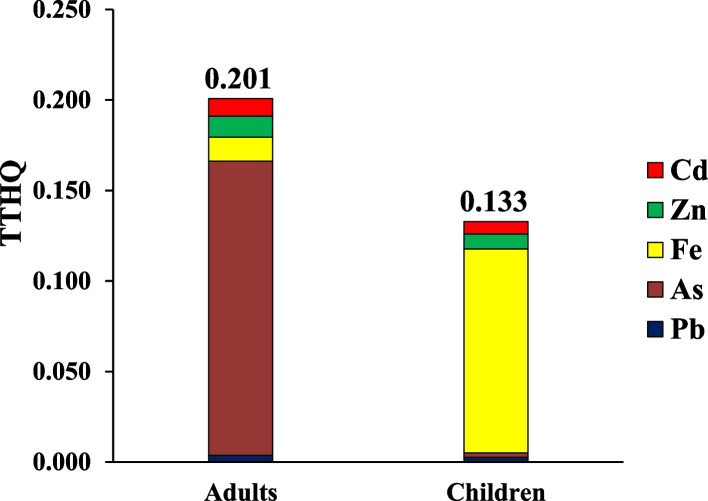
TTHQ in adults and children due to ingestion traditional vegetable oils content of PTEs

**Fig. 2:**
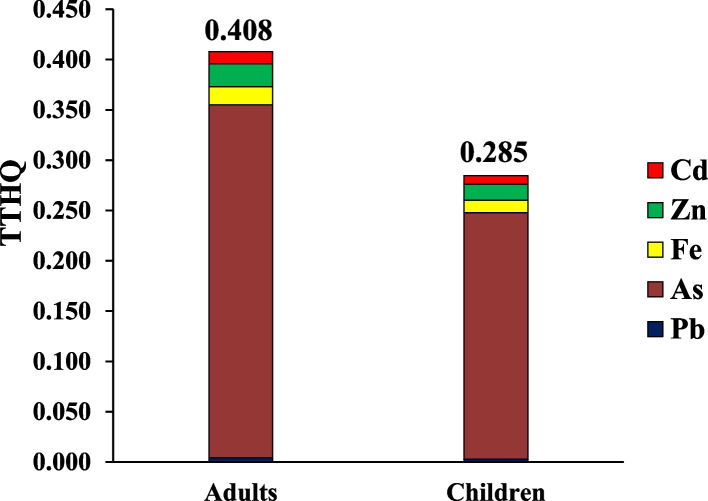
TTHQ in adults and children due to ingestion industrial vegetable oils content of PTEs

**Fig. 3:**
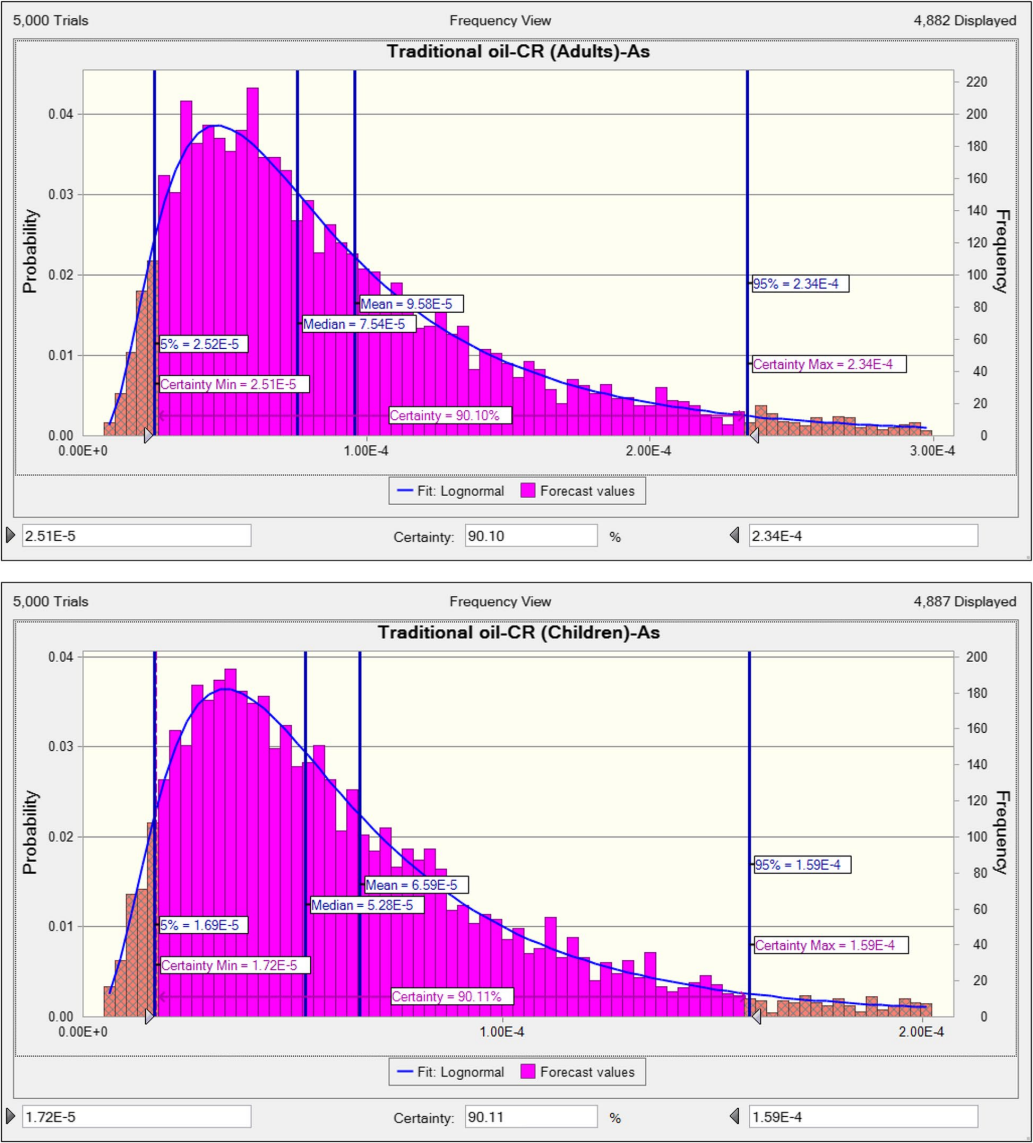
CR in adults and children due to ingestion traditional vegetable oils content of inorganic As

**Fig. 4:**
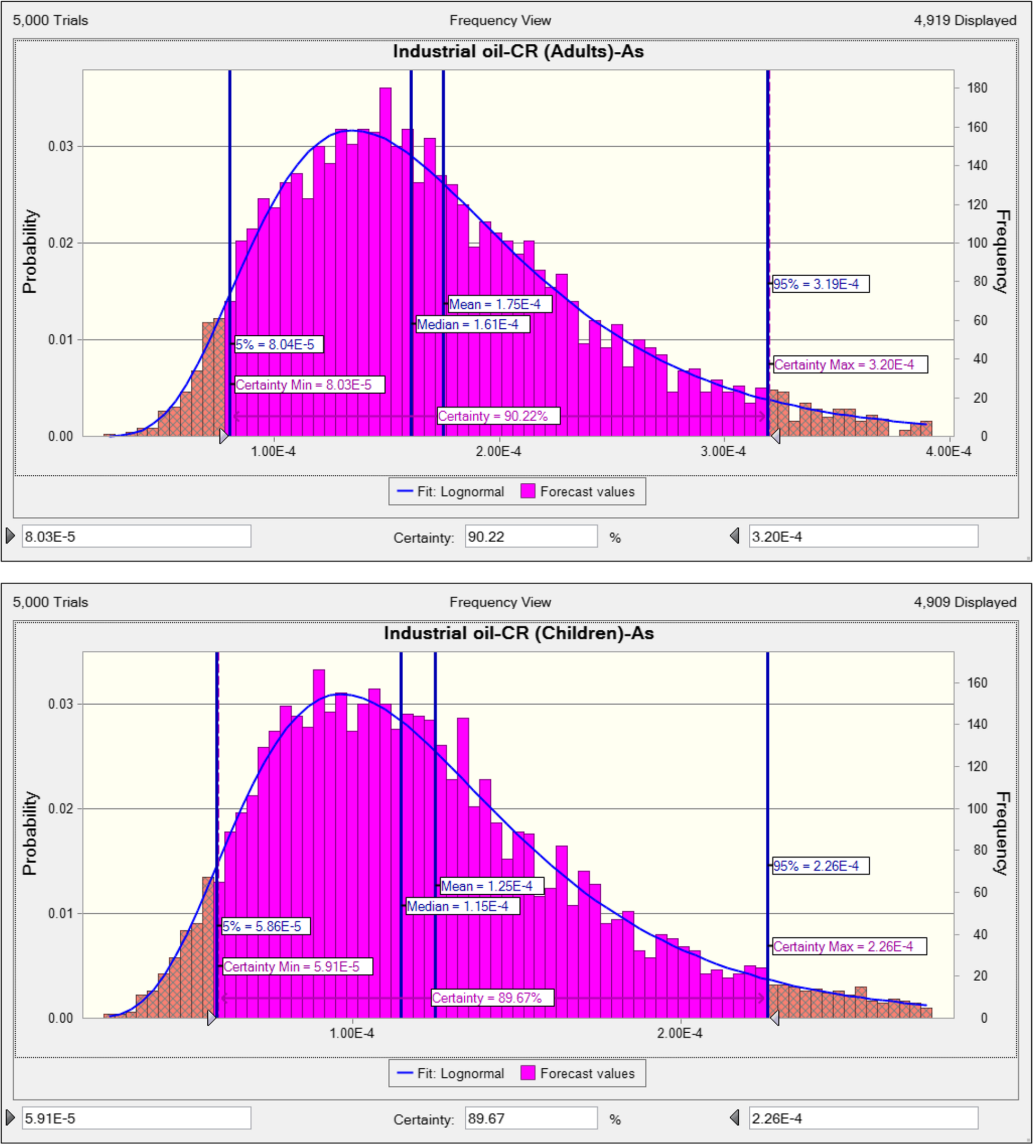
CR in adults and children due to ingestion industrial vegetable oils content of inorganic As

The exposure to little concentrations of PTEs can induce health complications for consumers. The loss of appetite, vomiting, diarrhea, and immune system disease, memory disorder, and damage the various tissue are one of the common complications of metal toxicity [98]. The rank order of PTEs based on their THQ in adults consumers due to ingestion traditional vegetable was As (0.1627) > Fe (0.0132) > Zn (0.0117) > Cd (0.0097) > Pb (0.0036) and in children, As (0.1128) > Fe (0.0092) > Zn (0.0082) > Cd (0.0068) > Pb (0.0025) (Appendix 1 and 2). TTHQ in adults and children due to ingestion traditional vegetable oils content of PTEs was equal to 0.201 and 0.133, respectively (Fig. 1). TTHQ in the both adults and children was lower than 1 value, hence consumer are at acceptable range due to ingestion traditional vegetable oils content of PTEs. The rank order of PTEs based on their THQ in adults consumers due to ingestion industrial vegetable was As (0.3510) Zn (0.0228) > Fe (0.0179) > Cd (0.0121) > Pb (0.0041) and in children, As (0.2450) > Zn (0.0159) > Fe (0.0125) > Cd (0.0084) > Pb (0.0029) (Appendix 3 and 4). TTHQ in adults and children due to ingestion industrial vegetable oils content of PTEs was equal to 0.408 and 0.285, respectively (Fig. 2). TTHQ in the both adults and children was lower than 1 value, hence consumer are at acceptable range (TTHQ < 1) due to ingestion industrial vegetable oils content of PTEs. In term of carcinogenic risk, as stated from investigations, median of CR in the adults and children due to ingestion traditional vegetable oils content of inorganic As was equal to 7.54E-5 and 5.28E-5, respectively (Fig. 3). CR the both adults and children was higher than safe range (CR < 1E-6), Hence consumer are at unacceptable risk due to ingestion traditional vegetable oils content of inorganic As. Median of CR in the adults and children due to ingestion industrial vegetable oils content of inorganic As was equal to 1.61E-4 and 1.15E-4, respectively (Fig. 4). CR the both adults and children was higher than safe range (CR < 1E-6), Hence consumer are at unacceptable risk due to ingestion industrial vegetable oils content of inorganic As. The risk pattern is different in countries and it can be related to agents including the content of PTEs, ingestion rate of vegetables oils, body weight and exposure time [99]. Consistent with our study, in study inducted by Haj Heidary et al. indicated the concentration of Pb, Cd, Zn and Fe in vegetables oils in Iran was safe and did not pose any health risks [81]. Zhu et al. reported that the concentration of PTEs (Cu, Zn, Fe, Cd, Pb and As) in vegetable oils was lesser than 1, therefore pose no risk to human health [84].

## Conclusion

This study was carried out to provide information on of PTEs (Cd, Pb, As, Fe, and Zn) content in traditional and industrial vegetable oils in Hamadan, West of Iran. According to the results, the highest and lowest level of PTEs found was related to Fe and Cd respectively. In samples investigated concentrations of PTEs in industrial vegetable oils were higher than traditional fruit juices *p* < 0.001. The content of Pb, Cd, As, Zn and Fe in all samples were detected to be lower compared the suggested legal limits. In term of non-carcinogenic, consumer were at acceptable range (TTHQ < 1) due to ingestion traditional and industrial vegetable oils content of PTEs. In term of carcinogenic, CR the both adults and children was higher than safe range (CR < 1.00E-6), Hence consumer are at unacceptable risk due to ingestion industrial vegetable oils content of inorganic As. Because of discharge of pollutants into the environment from some resources, including, industrial and agricultural activities, stricter inspection and control on vegetable oils should be enforced. Moreover, evaluating of the effect of different production processes on the content of metals in vegetable oils is recommended.

### Supplementary Information

**Additional file 1:** **Appendix 1.** THQ in the adults consumers due to ingestion traditional vegetable oils content of PTEs. **Appendix 2.** THQ in the children consumers due to ingestion traditional vegetable oils content of PTEs. **Appendix 3.** THQ in the adults consumers due to ingestion industrial vegetable oils content of PTEs. **Appendix4.** THQ in the children consumers due to ingestion industrial vegetable oils content of PTEs.

## Data Availability

The datasets used and/or analysed during the current study available from the corresponding author on reasonable request.
