# Supplementary material for: A probabilistic health risk assessment of potentially toxic elements in edible vegetable oils consumed in Hamadan, Iran
Source: BMC Public Health. 2024 Jan 18;24:218. doi: 10.1186/s12889-023-17624-1 (PMC10797719; doi:10.1186/s12889-023-17624-1)
Supplement: Supplementary file 1 — Additional file 1: Appendix 1. THQ in the adults consumers due to ingestion traditional vegetable oils content of PTEs. Appendix 2. THQ in the children consumers due to ingestion traditional vegetable oils content of PTEs. Appendix 3. THQ in the adults consumers due to ingestion industrial vegetable oils content of PTEs. Appendix4. THQ in the children consumers due to ingestion industrial vegetable oils content of PTEs. [file 12889_2023_17624_MOESM1_ESM.docx]

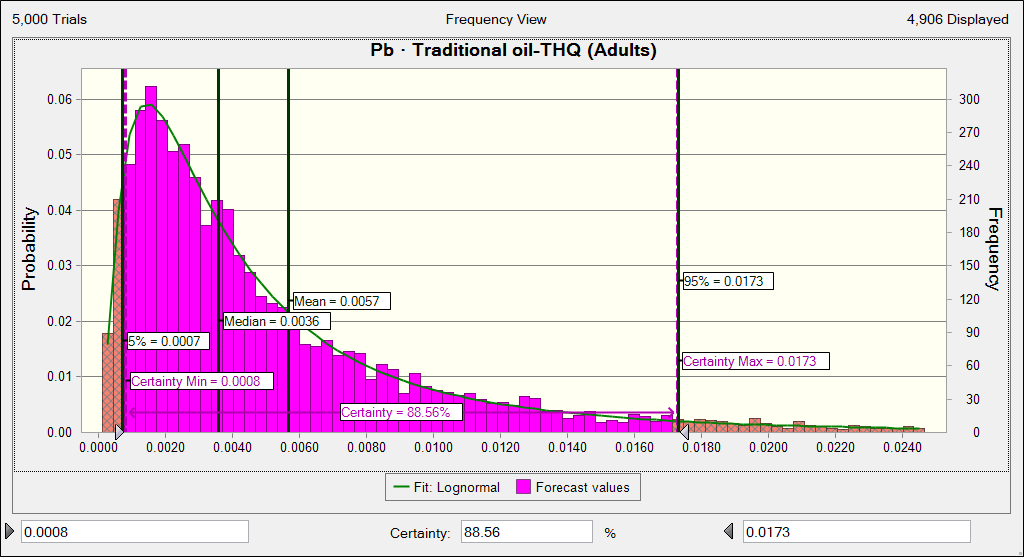


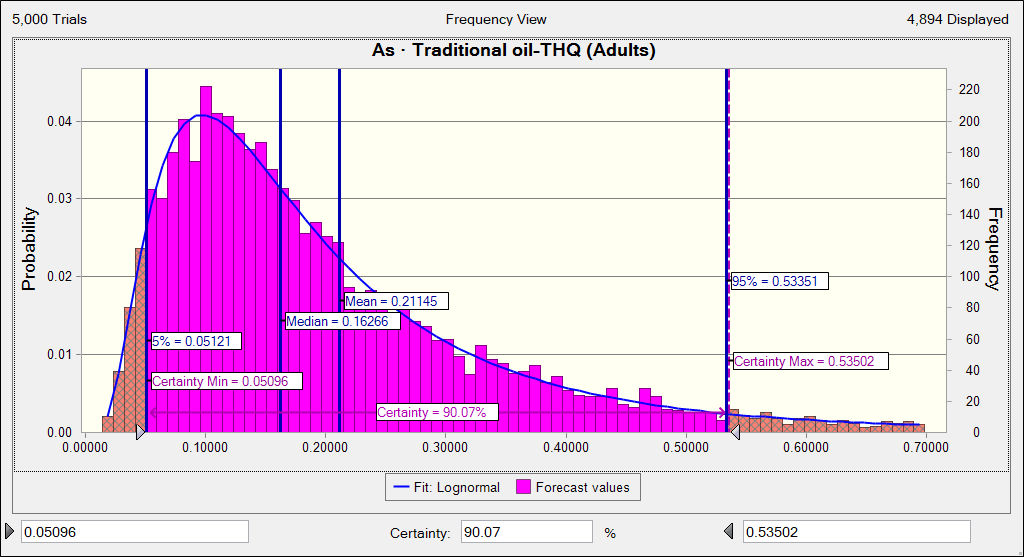


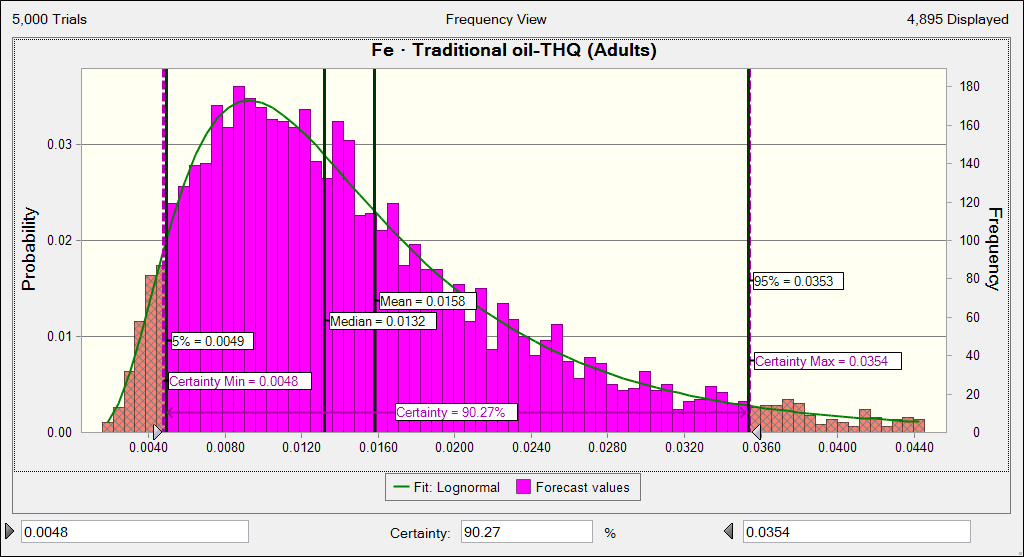


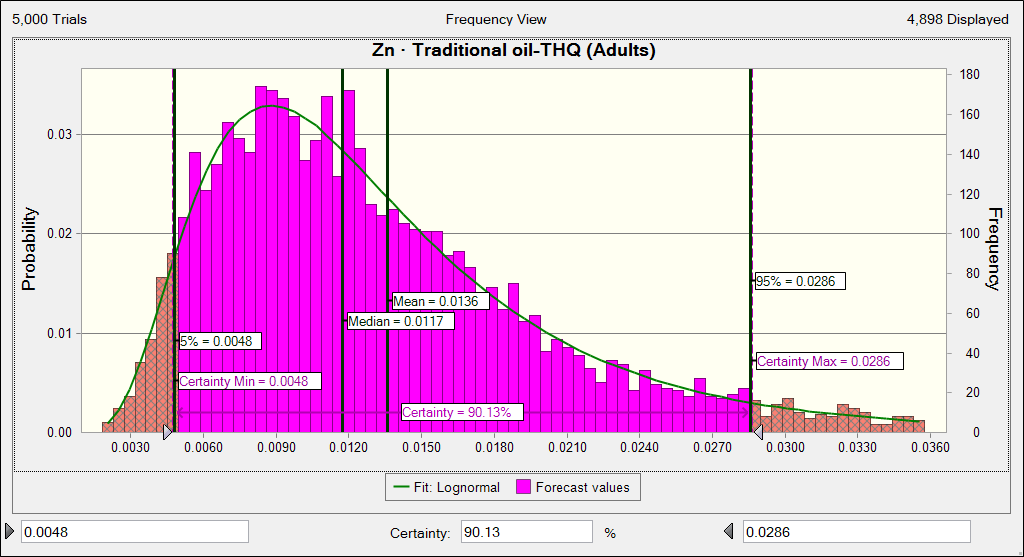


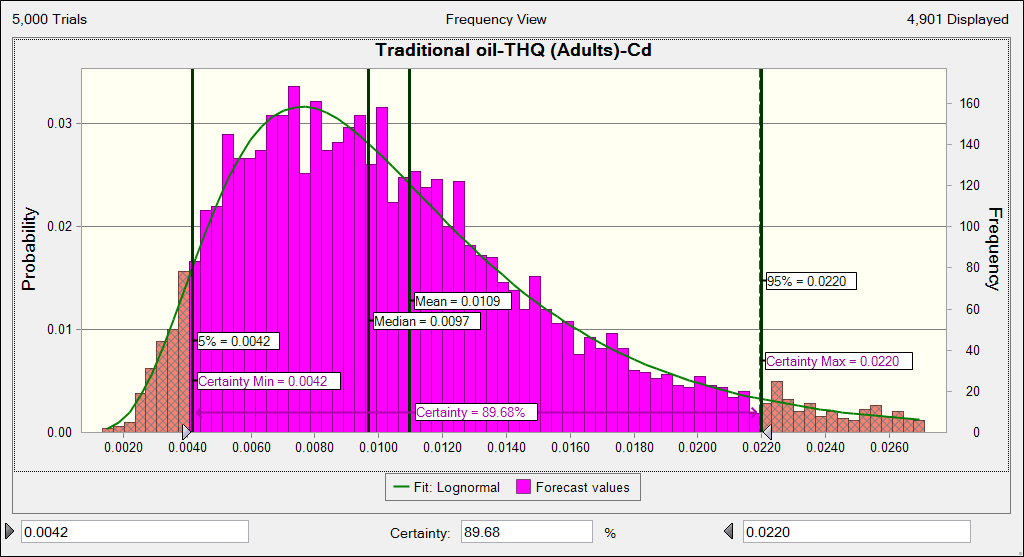


**Appendix 1.** THQ in the adults consumers due to ingestion traditional vegetable oils content of PTEs


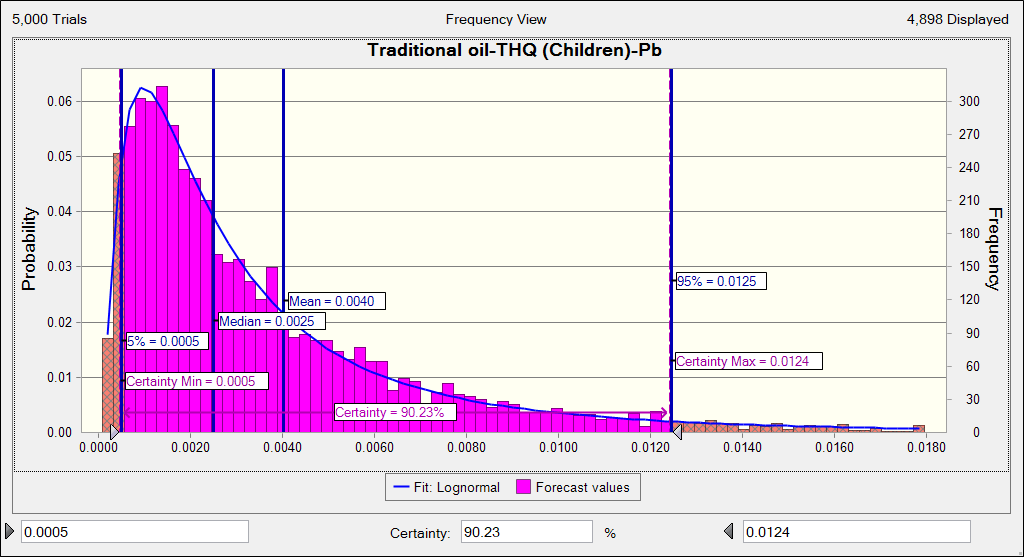


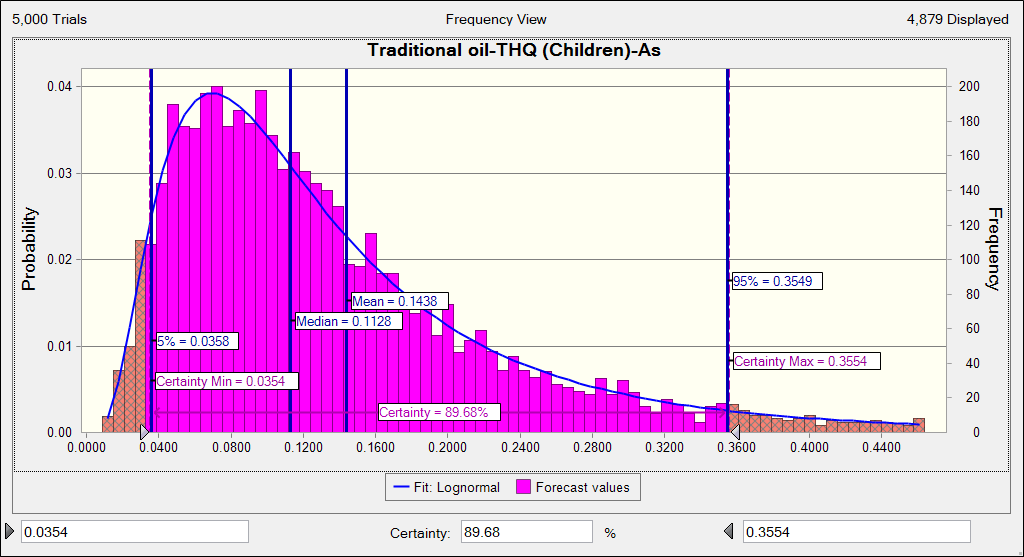


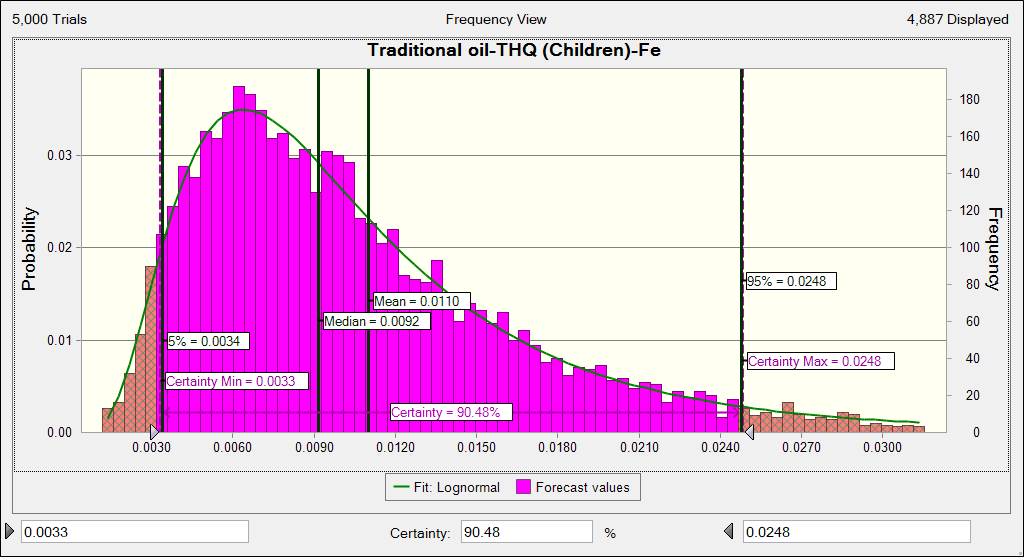


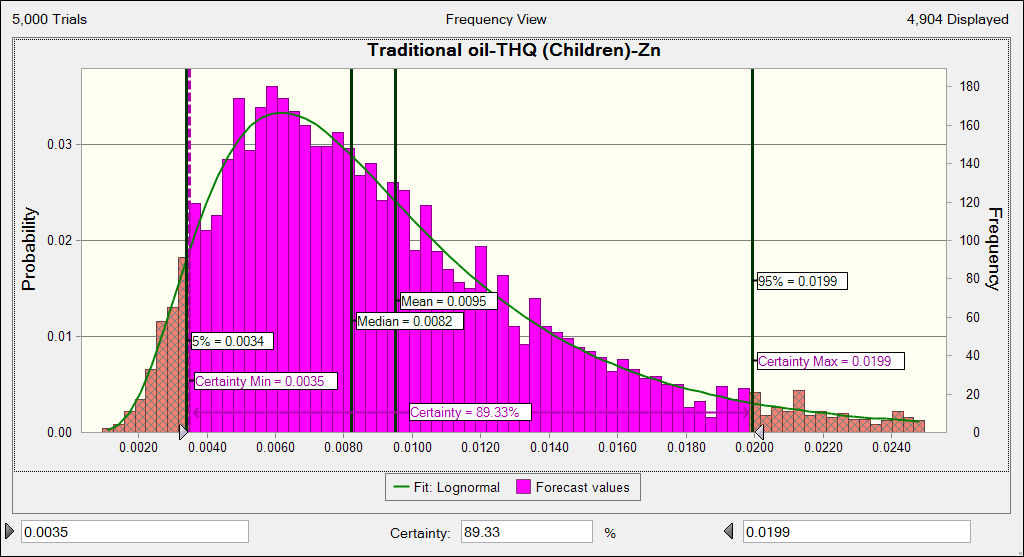


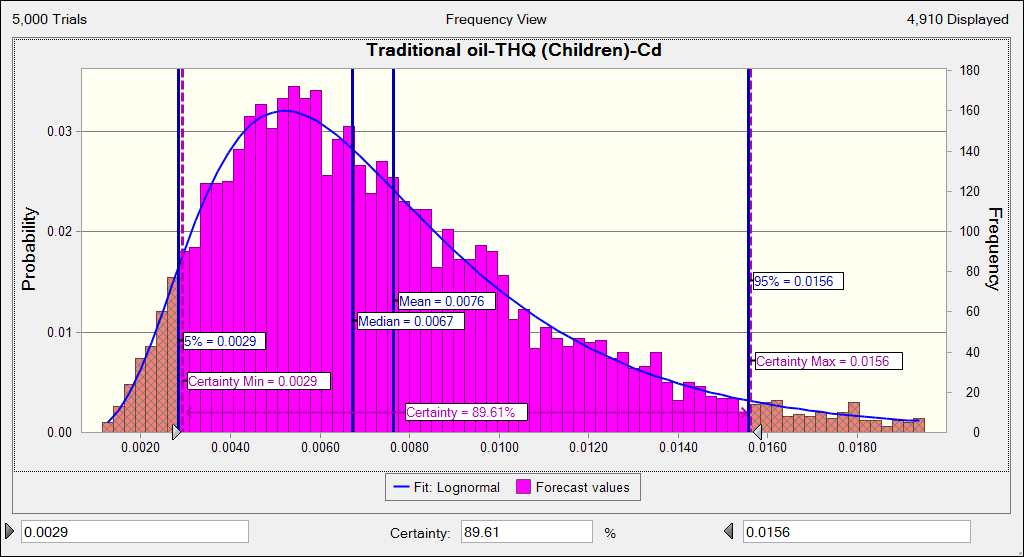


**Appendix 2.** THQ in the children consumers due to ingestion traditional vegetable oils content of PTEs


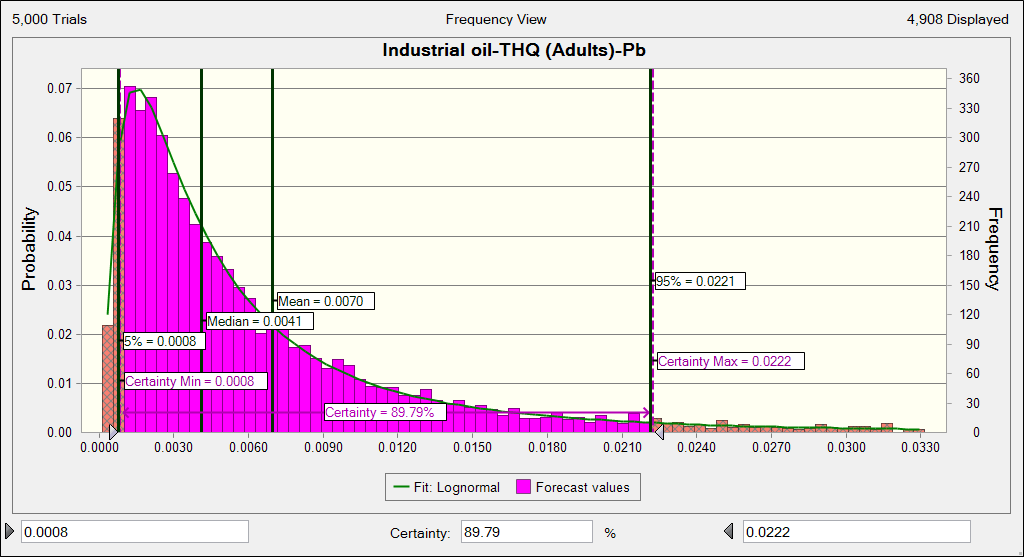


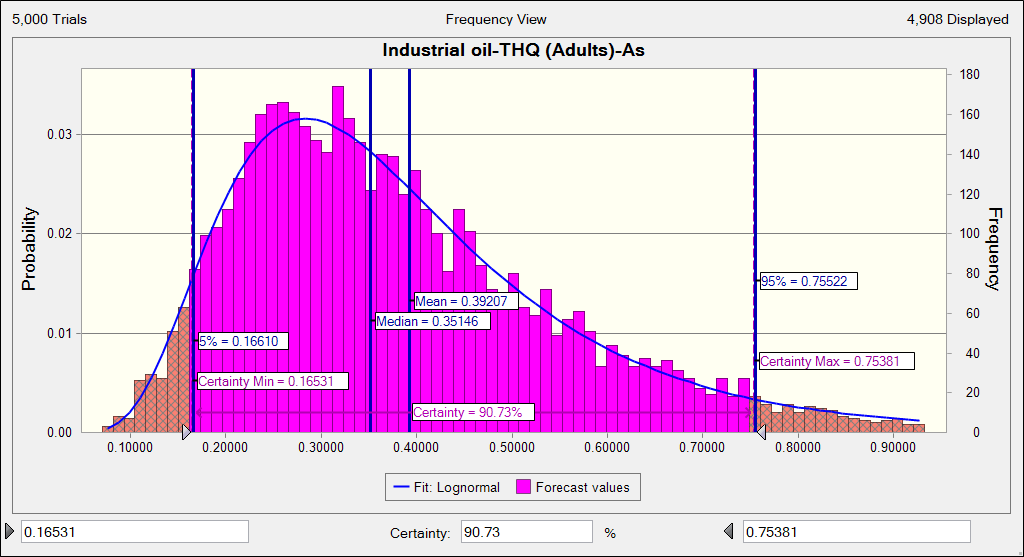


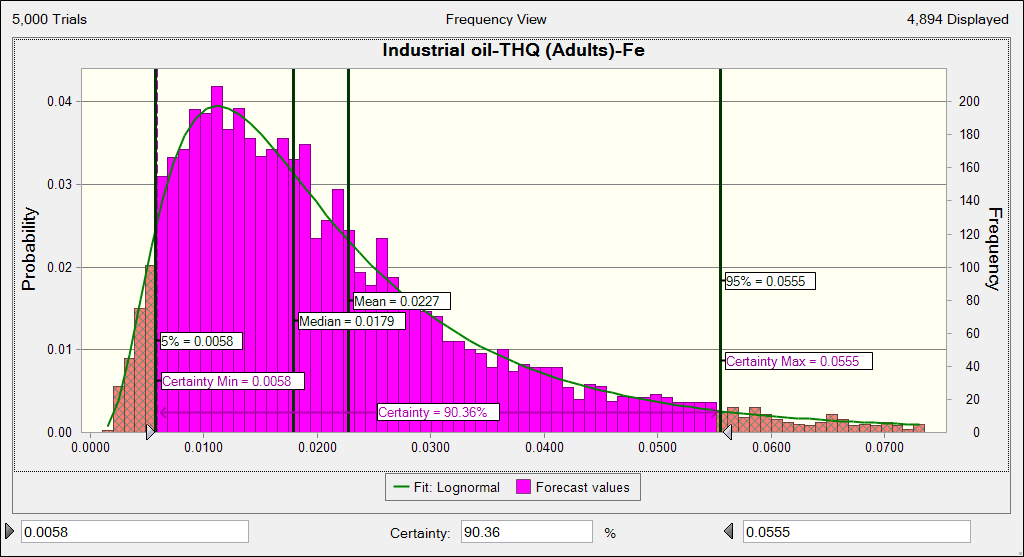


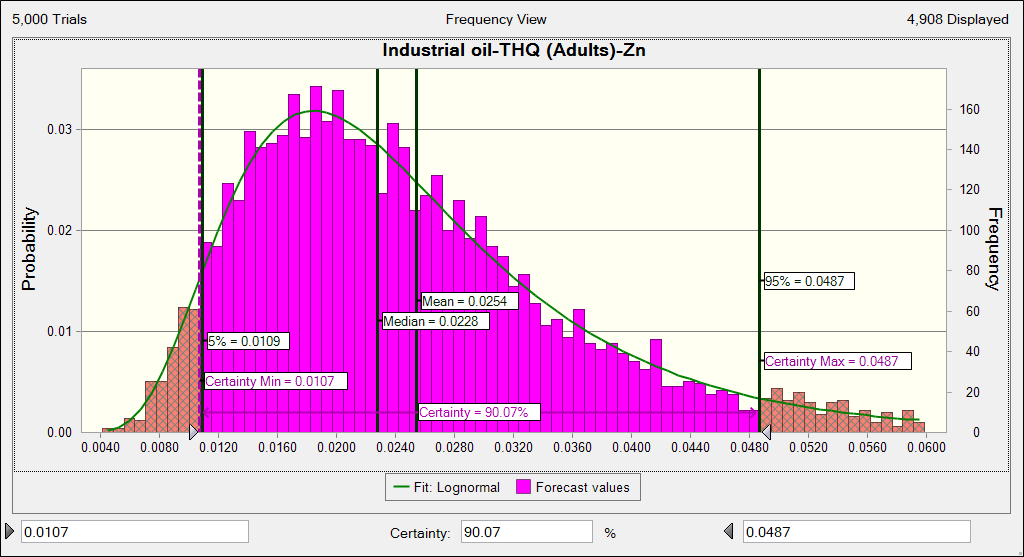


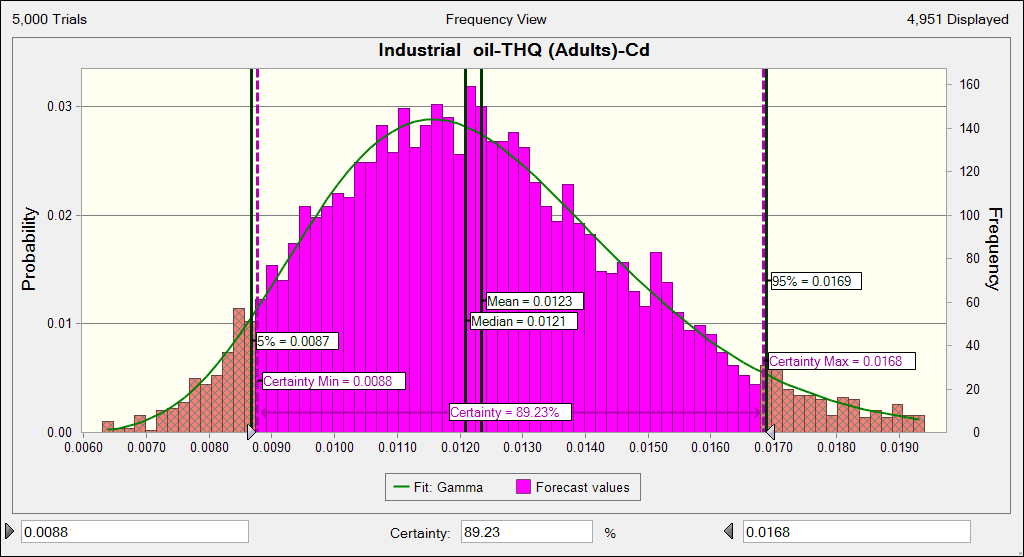


**Appendix 3.** THQ in the adults consumers due to ingestion industrial vegetable oils content of PTEs


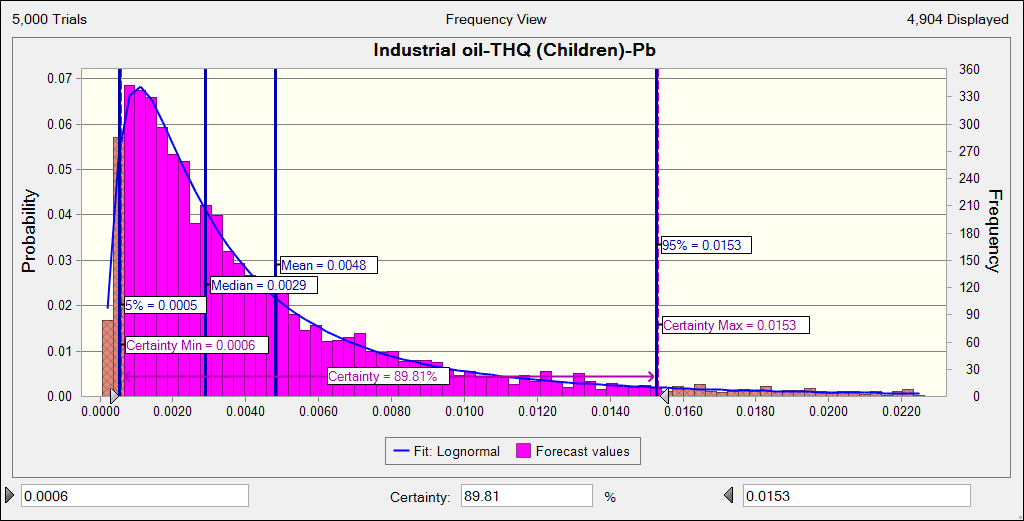


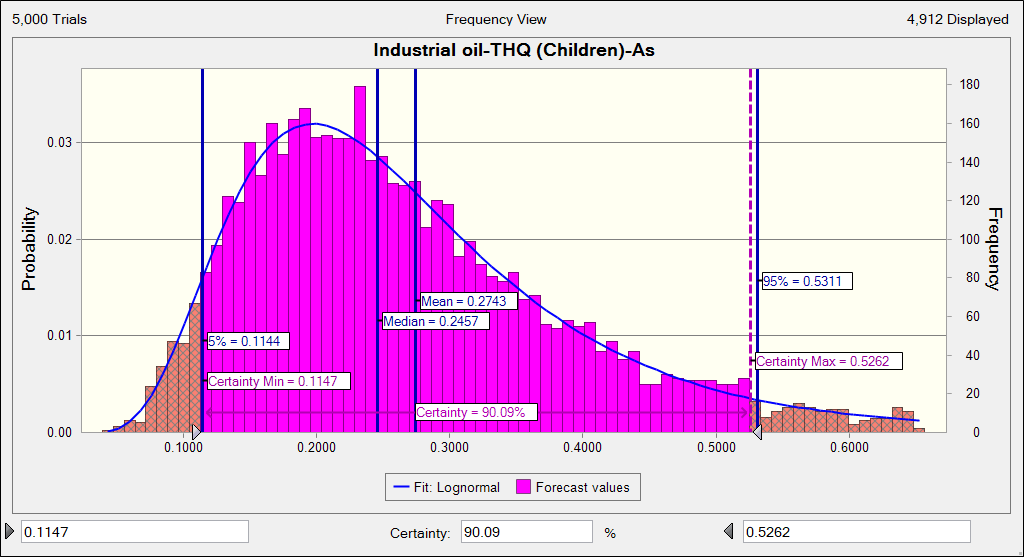


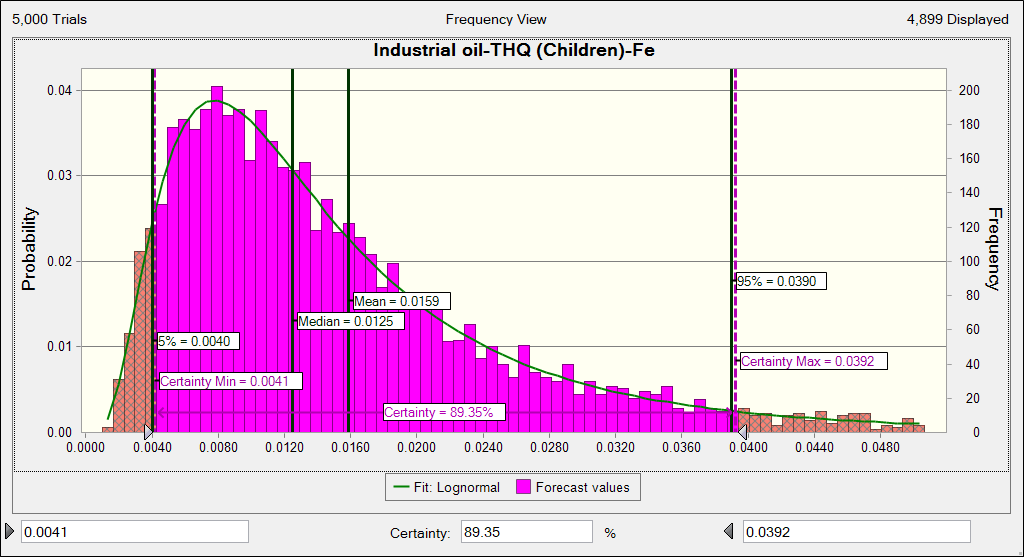


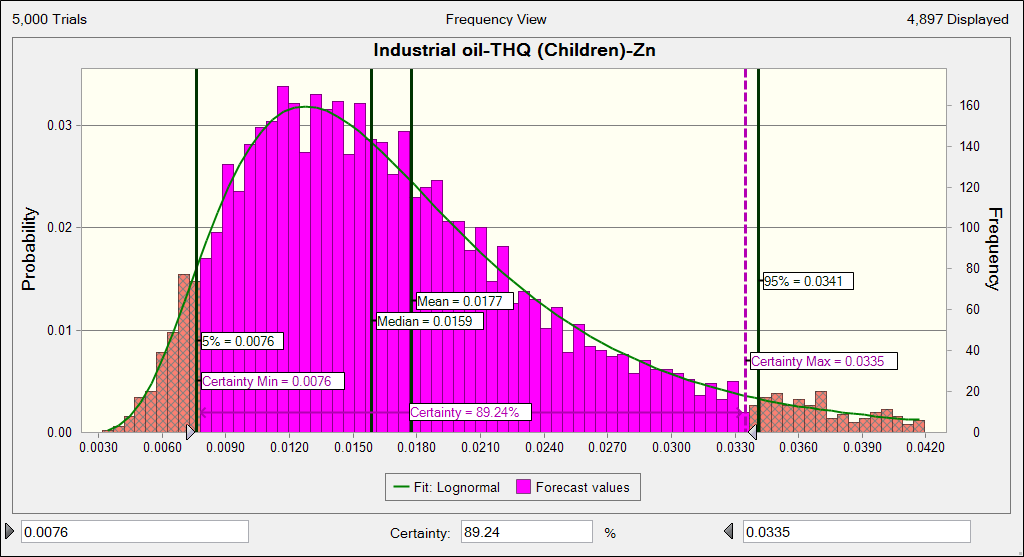


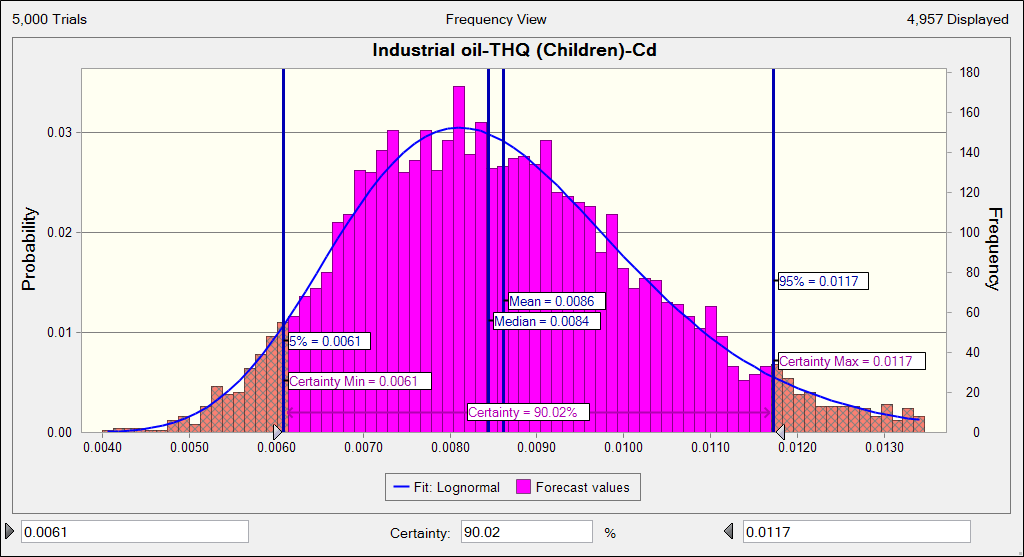


**Appendix4.** THQ in the children consumers due to ingestion industrial vegetable oils content of PTEs
